# Experimental Results of Attitude Determination Functional Algorithms Implementation in Strapdown Inertial Navigation System

**DOI:** 10.3390/s22051849

**Published:** 2022-02-26

**Authors:** Maksim Zharkov, Konstantin Veremeenko, Ivan Kuznetsov, Andrei Pronkin

**Affiliations:** Automated Complexes of Attitude and Navigation Systems Department, Moscow Aviation Institute (National Research University), 125993 Moscow, Russia; nio3@mai.ru (K.V.); im_kuznetsov@mai.ru (I.K.); an_pronkin@mai.ru (A.P.)

**Keywords:** attitude determination, strapdown inertial navigation system, attitude and heading reference system

## Abstract

Strapdown inertial navigation system (SINS) software developers are usually mainly focused on the attitude determination algorithm design and characteristics. Such an algorithm can be based on different mathematical apparatus. An approach to the derivation of attitude determination algorithm equations in strapdown inertial navigation system is proposed. This algorithm is based on direct heading, pitch, and roll calculation. The qualitative differences between the proposed algorithm and the one using a transformation matrix are noted. The main objective of the paper is direct attitude angles calculation algorithm application possibilities analysis in real SINS operating conditions. The analysis is based on a comparison of the proposed algorithm with another frequently used attitude determination algorithm based on the transformation matrix. Attitude determination algorithms performance investigation results based on car and helicopter experimental tests are presented.

## 1. Introduction

Strapdown inertial navigation systems (SINS) are widely used in various applications [1,2,3,4,5,6,7]. The key task of SINS design is the development of attitude determination algorithm providing attitude angles (heading, pitch, and roll) calculation within required accuracy and output rate [8]. Currently, many types of attitude determination algorithms have been developed on the base of the transformation matrix, Euler angles, quaternions, or rotation vector.

From a mathematical point of view, all these parameters lead to the same results for describing the algorithms of the SINS operation. The kinematic equations of quaternion parameters are linear, have the fourth order, and are defined for any yaw, pitch, and roll angles. The quaternion parameters require only one normalization equation. Kinematic equations in Euler angles have a low order (the third) and a clear structure, but contain trigonometric functions from the desired angles and allow singularity at a pitch angle equals to 90 degrees. The SINS attitude equations written using a transformation matrix are linear, defined for any yaw, pitch, and roll angles, but the equations have a fairly high order (the ninth). In addition, the equations must be supplemented with six coupling equations. Taking this into account, quaternions have been preferred for a long time in the numerical SINS attitude algorithms implementation [8,9,10,11].

The Moscow Aviation Institute MAI has been carrying out research and development in the field of INS since the middle of the last century. Currently, MAI has created a line of various accuracy grade SINS, depending on the type of gyroscopes used: from MEMS to laser gyroscopes [2,3]. Due to the increasing capabilities of embedded computers in the latest modifications of SINS, the attitude algorithm is based on a transformation matrix. These types of algorithms have such disadvantages as errors of scale and non-orthogonality growth and high-level (ninth) order of the system differential equations requiring a solution. It is possible to get rid of these disadvantages with the use of a transformation matrix scaling an orthogonalization special procedures [8,9,10,11] and the use of higher performance computers. At the same time, these disadvantages can also be eliminated by switching from the algorithm for calculating the elements of the transformation matrix to an algorithm of direct heading, pitch, and roll angles (Euler angles) calculation.

In this regard, the main objective of the paper is the analysis of direct attitude angles calculation algorithm practical application possibilities in various grades SINS using modern hardware onboard different types of mobile objects. To carry out such an analysis, a possible approach to the derivation of the direct heading, pitch, and roll angles calculation algorithm equations is proposed. The analysis is based on a comparison of the direct angles calculation algorithm with the transformation matrix based algorithm when they are implemented in the INS computer during car and helicopter tests.

It should be noted that the direct angles calculation algorithm has not been compared with a quaternions-based algorithm, since the quaternions-based algorithm research results have been published by many authors [8,9,10,11]. The accuracy and computational efficiency of the quaternions-based algorithm should, theoretically, be comparable to the direct angles calculation algorithm. However, additional calculations using inverse trigonometric functions will be required to obtain heading, pitch, and roll by the quaternions values.

The paper is organized as follows. In Section 2, the mathematical description of attitude parameters being used, widely applicable transformation matrix algorithm basic equations and algorithm of direct heading, pitch, and roll angles calculation basic equation derivation, is given. In Section 3 the technique, the main providing conditions, and the results of experimental studies of different types of attitude algorithms on different types of SINS installed on a car and helicopter, are given. Since the Global Navigation Satellite System (GNSS) receiver data was available on board during the experiments, attitude algorithms were implemented during the tests using high-speed measurements of the GNSS receiver. The test results of the SINS attitude algorithms based only on inertial measurements are compared with the attitude algorithms including high-speed measurements of the GNSS receiver. Analysis of the results of the experimental study are given in Section 4.

## 2. Direct Attitude Angles Calculations

In this paper, heading, pitch, and roll attitude parameters, which determine the rotations of the body frame relative to the local level frame, are being considered [12]. Unit vectors ${\bar{i}}_{b}$, ${\bar{j}}_{b}$, and ${\bar{k}}_{b}$ implement the body frame, and $\bar{i}$ (East), $\bar{j}$ (North), and $\bar{r}$ (Up), the local level frame. Initialization conditions are: ${\bar{i}}_{b0}=\bar{j}$, ${\bar{j}}_{b0}=\bar{r}$, and ${\bar{k}}_{b0}=\bar{i}$.

**Figure 1:**
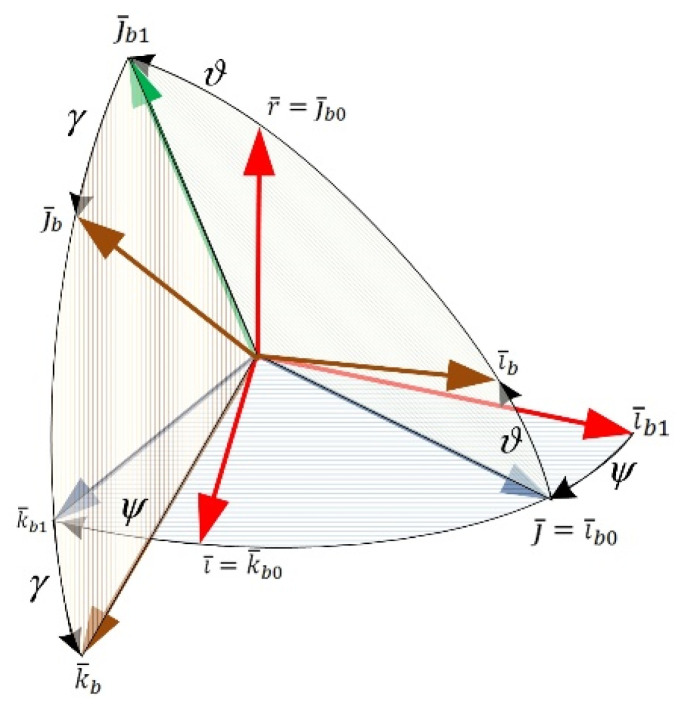
Frames and unit vectors.

Body frame rotation from the initial position is performed by following the sequence of rotations. The first rotation is about the axis including the unit vector $\bar{r}$ through the ψ angle clockwise. In this case, the unit vectors ${\bar{i}}_{b0}$ and ${\bar{k}}_{b0}$ will move to the ${\bar{i}}_{b1}$ and ${\bar{k}}_{b1}$ positions (shown by a dotted line in the Figure 1). The second rotation is about the axis including the unit vector ${\bar{k}}_{b1}$ through the pitch angle ϑ. As a result, the unit vectors ${\bar{i}}_{b1}$ and ${\bar{j}}_{b0}$ will move to the ${\bar{i}}_{b}$ and ${\bar{j}}_{b1}$ positions. Finally, the rotation about the roll angle *γ* occurs through the axis including the unit vector ${\bar{i}}_{b}$. The final rotation leads to the updated axes positions ${\bar{i}}_{b}$, ${\bar{j}}_{b}$, and ${\bar{k}}_{b}$, and the body frame will be turned through the heading, pitch, and roll from its initial position. The vectors $\bar{\dot{\psi }}$, $\bar{\dot{\vartheta }}$, and $\bar{\dot{\gamma }}$ corresponding to heading, pitch, and roll angular rates are presented by the following form:

$$\bar{\dot{\psi }}=-\bar{r}\dot{\psi },\;\bar{\dot{\vartheta }}={\bar{k}}_{b1}\dot{\vartheta },\;\bar{\dot{\gamma }}={\bar{i}}_{b}\dot{\gamma }.$$

The unit vectors ${\bar{k}}_{b1}$ and ${\bar{i}}_{b}$ using $\bar{i}$, $\bar{j}$, and $\bar{r}$ basis can be defined in terms of (Figure 1):

$$\begin{array}{c} {\bar{k}}_{b1}=\bar{i}\cos\psi -\bar{j}\sin\psi ,\\ {\bar{i}}_{b}=\bar{i}\sin\psi \cos\vartheta +\bar{j}\cos\psi \cos\vartheta +\bar{r}\sin\vartheta . \end{array}$$

Now the sum of the vectors $\bar{\dot{\psi }}$, $\bar{\dot{\vartheta }}$, and $\bar{\dot{\gamma }}$ can be written as follows:

(1) $$\bar{\dot{\psi }}+\bar{\dot{\vartheta }}+\bar{\dot{\gamma }}=\bar{i}\left(\dot{\vartheta }\cos\psi +\dot{\gamma }\sin\psi \cos\vartheta \right)+\bar{j}\left(-\dot{\vartheta }\sin\psi +\dot{\gamma }\cos\psi \cos\vartheta \right)+\bar{r}\left(-\dot{\psi }+\dot{\gamma }\sin\vartheta \right).$$

The direction cosine matrix relating $\bar{i}$, $\bar{j}$, and $\bar{r}$ basis, and ${\bar{i}}_{b}$, ${\bar{j}}_{b}$, and ${\bar{k}}_{b}$ basis can be denoted as $\left[C_{ij}\right]$. The elements of the $\left[C_{ij}\right]$ matrix in terms of ψ, ϑ, and γ angles can be expressed as:

(2) $$\left[C_{ij}\right]=\left[\begin{array}{ccc} \sin\psi \cos\vartheta & \cos\psi \sin\gamma -\sin\psi \cos\gamma \sin\vartheta & \cos\psi \cos\gamma +\sin\psi \sin\vartheta \sin\gamma \\ \cos\psi \cos\vartheta & -\sin\psi \sin\gamma -\cos\psi \sin\vartheta \cos\gamma & -\sin\psi \cos\gamma +\cos\psi \sin\vartheta \sin\gamma \\ \sin\vartheta & \cos\vartheta \cos\gamma & -\cos\vartheta \sin\gamma \end{array}\right]$$

In case of attitude determination functional algorithm, onboard computer (OC) implementation matrix $\left[C_{ij}\right]$ would be determined by solving differential equation:

(3) $$\frac{d\left[C_{ij}\right]}{dt}=\left[\Omega_{l}\right]\left[C_{ij}\right]-\left[C_{ij}\right]\left[\Omega_{b}\right]$$

In Equation (3):

$$\left[\Omega_{l}\right]=\left[\begin{array}{ccc} 0 & \Omega_{Z} & -\Omega_{Y}\\ -\Omega_{Z} & 0 & \Omega_{X}\\ \Omega_{Y} & -\Omega_{X} & 0 \end{array}\right],$$

$$\left[\Omega_{b}\right]=\left[\begin{array}{ccc} 0 & \Omega_{3} & -\Omega_{2}\\ -\Omega_{3} & 0 & \Omega_{1}\\ \Omega_{2} & -\Omega_{1} & 0 \end{array}\right],$$

where $\Omega_{X,Y,Z}$ are local level frame absolute angular rate vector ${\;\bar{\Omega }}_{l}$ projections on to the local level frame axis with $\bar{i}$, $\bar{j}$, and $\bar{r}$ unit vectors; and $\Omega_{1,2,3}$ are body frame absolute angular rate vector ${\;\bar{\Omega }}_{b}$ projections on to the body frame axis with ${\bar{i}}_{b}$, ${\bar{j}}_{b}$, and ${\bar{k}}_{b}$ unit vectors.

Solving the Equation (3) requires specification of the initial values of the $\left[C_{ij}\right]$ matrix. At the same time, $\Omega_{1,2,3}$ values are received by OC from gyros unit and $\Omega_{x,y,z}$ values are computed in OC during navigation algorithm implementation.

In turn, navigation algorithm can be implemented if the following operation is being performed as a preliminary:

(4) $$\left[\begin{array}{c} n_{X}\\ n_{Y}\\ n_{Z} \end{array}\right]=\left[C_{ij}\right]\left[\begin{array}{c} n_{1}\\ n_{2}\\ n_{3} \end{array}\right],$$

where $n_{1,2,3}$ are acceleration vector $\bar{n}$ projections on to the body frame axis (values are received by OC from accelerometers unit); and $n_{X,Y,Z}$ are acceleration vector $\bar{n}$ projections on to the local level frame axis.

In other sources attitude determination algorithms based on quaternions are also being considered. These algorithms differ from those considered above only by the $\left[C_{ij}\right]$ transformation matrix computing setup approach. The $\left[C_{ij}\right]$ matrix is computed using a direction cosine matrix related to body frame and reference (inertial) frame by unit vectors $\bar{\xi }$, $\bar{\eta }$, $\bar{\zeta }$:

$$\left[\begin{array}{c} \bar{\xi }\\ \bar{\eta }\\ \bar{\varsigma } \end{array}\right]=\left[A_{ij}\right]\left[\begin{array}{c} {\bar{i}}_{b}\\ {\bar{j}}_{b}\\ {\bar{k}}_{b} \end{array}\right]$$

and a direction cosine matrix related to local level frame and the same reference (inertial) frame:

$$\left[\begin{array}{c} \bar{\xi }\\ \bar{\eta }\\ \bar{\varsigma } \end{array}\right]=\left[B_{ij}\right]\left[\begin{array}{c} \bar{i}\\ \bar{j}\\ \bar{r} \end{array}\right]$$

Matrix $\left[C_{ij}\right]$ can be determined as follows:

$$\left[C_{ij}\right]={\left[B_{ij}\right]}^{T}\left[A_{ij}\right].$$

In turn, to determine $\left[A_{ij}\right]$ and $\left[B_{ij}\right]$ matrices, linear systems of differential equations are used, each one of it is of the fourth order, and the unknowns are the elements of the corresponding quaternions. ψ, ϑ, and γ angles are still determined from the $\left[C_{ij}\right]$ matrix elements. $\left[A_{ij}\right]$ and $\left[B_{ij}\right]$ matrices can also be determined by solving differential equations systems as follows:

(5) $$\begin{array}{c} \frac{d\left[A_{ij}\right]}{dt}=\left[A_{ij}\right]\left[\begin{array}{ccc} 0 & \Omega_{3} & -\Omega_{2}\\ -\Omega_{3} & 0 & \Omega_{1}\\ \Omega_{2} & -\Omega_{1} & 0 \end{array}\right],\\ \frac{d\left[B_{ij}\right]}{dt}=\left[B_{ij}\right]\left[\begin{array}{ccc} 0 & \Omega_{Z} & -\Omega_{Y}\\ -\Omega_{Z} & 0 & \Omega_{X}\\ \Omega_{Y} & -\Omega_{X} & 0 \end{array}\right]. \end{array}$$

It seems practically important to develop an algorithm of direct ψ, ϑ, and γ angles calculation, instead of a calculation using another attitude parameters.

The way in which direct attitude angles calculations algorithm can be developed is shown below [12].

${\;\bar{\Omega }}_{b}$ body frame absolute angular rate vector can be presented as follows:

(6) $${\bar{\Omega }}_{b}={\;\bar{\Omega }}_{l}+\bar{\dot{\psi }}+\bar{\dot{\vartheta }}+\bar{\dot{\gamma }},$$

where ${\;\bar{\Omega }}_{l}$ is the local level frame absolute angular rate vector.

On the other hand, ${\bar{\Omega }}_{b}$ vector can be written as follows:

(7) $${\;\bar{\Omega }}_{b}={\bar{i}}_{b}\Omega_{1}+{\bar{j}}_{b}\Omega_{2}+{\bar{k}}_{b}\Omega_{3}$$

and ${\;\bar{\Omega }}_{l}$ vector using unit vectors $\bar{i}$, $\bar{j}$, and $\bar{r}$ can be written as follows:

$${\;\bar{\Omega }}_{l}={\bar{i}}_{b}\Omega_{X}+{\bar{j}}_{b}\Omega_{Y}+{\bar{k}}_{b}\Omega_{Z}$$

where $\Omega_{1,2,3}$ are body frame absolute angular rate projections on to the body frame axis;

(8) $$\begin{array}{c} \Omega_{X}=-\dot{\varphi },\\ \Omega_{Y}=\left(u+\dot{\lambda }\right)\cos\varphi ,\\ \Omega_{Z}=\left(u+\dot{\lambda }\right)\sin\varphi , \end{array}$$

u is the Earth angular rate, $\dot{\varphi }$ is the latitude angular rate, and $\dot{\lambda }$ is the longitude angular rate.

Heading, pitch, and roll angular rate vectors in (6) can be written using unit vectors $\bar{i}$, $\bar{j}$, and $\bar{r}$:

(9) $$\bar{\dot{\psi }}=-\bar{r}\dot{\psi },$$

(10) $$\bar{\dot{\vartheta }}=\bar{i}\;\dot{\vartheta }\cos\psi -\bar{j}\;\dot{\vartheta }\sin\psi ,$$

(11) $$\bar{\dot{\gamma }}=\bar{i}\;\dot{\gamma }\sin\psi \cos\vartheta +\bar{j}\;\dot{\gamma }\cos\psi \cos\vartheta +\bar{r}\;\dot{\gamma }\sin\vartheta .$$

The sign “−“ in (9) takes into account the fact of clockwise heading count.

Now, taking into account expressions (7)–(11), the vector equality (6) can be represented as:

(12) $$\begin{array}{c} \bar{i}\left(\dot{\vartheta }\cos\psi +\dot{\gamma }\sin\psi \cos\vartheta \right)+\bar{j}\left(-\dot{\vartheta }\sin\psi +\dot{\gamma }\cos\psi \cos\vartheta \right)+\bar{r}\left(-\dot{\psi }+\dot{\gamma }\sin\vartheta \right)\\ ={\bar{i}}_{b}\Omega_{1}+{\bar{j}}_{b}\Omega_{2}+{\bar{k}}_{b}\Omega_{3}+\bar{i}\;\dot{\varphi }-\bar{j}\cdot \left(u+\dot{\lambda }\right)\cdot \cos\varphi -\bar{r}\left(u+\dot{\lambda }\right)\sin\varphi . \end{array}$$

Multiplying the left and right sides of this vector equality scalarly and sequentially by unit vectors $\bar{i}$, $\bar{j}$, and $\bar{r}$, the following equation can be obtained:

(13) $$\left[\begin{array}{ccc} 0 & \cos\psi & \sin\psi \cos\vartheta \\ 0 & -\sin\psi & \cos\psi \cos\vartheta \\ -1 & 0 & \sin\vartheta \end{array}\right]\left[\begin{array}{c} \dot{\psi }\\ \dot{\vartheta }\\ \dot{\gamma } \end{array}\right]=\left[\begin{array}{c} \bar{i}\cdot {\;\bar{\Omega }}_{b}+\dot{\varphi }\\ \bar{j}\cdot {\;\bar{\Omega }}_{b}-\left(u+\dot{\lambda }\right)\cos\varphi \\ \bar{r}\cdot {\;\bar{\Omega }}_{b}-\left(u+\dot{\lambda }\right)\sin\varphi \end{array}\right].$$

Denote the square matrix on the left side of expression (13) as $\left[M_{ij}\right]$. The inverse matrix ${\left[M_{ij}\right]}^{-1}$ has the form:

(14) $${\left[M_{ij}\right]}^{-1}=\left[\begin{array}{ccc} \sin\psi \tan\vartheta & \cos\psi \tan\vartheta & -1\\ \cos\psi & -\sin\psi & 0\\ \frac{\sin\psi }{\cos\vartheta } & \frac{\cos\psi }{\cos\vartheta } & 0 \end{array}\right].$$

Considering ${\left[\begin{array}{ccc} \bar{i}\;{\bar{\Omega }}_{b} & \bar{j}{\bar{\Omega }}_{b} & \bar{r}{\;\bar{\Omega }}_{b} \end{array}\right]}^{T}=\left[C_{ij}\right]\cdot {\left[\begin{array}{ccc} \Omega_{1} & \Omega_{2} & \Omega_{3} \end{array}\right]}^{T}$ and taking into account the $\left[C_{ij}\right]$ matrix has the form (2), after left multiplying (13) by the matrix ${\left[M_{ij}\right]}^{-1}$ the following result can be obtained:

(15) $$\frac{d}{dt}\left[\begin{array}{c} \psi \\ \vartheta \\ \gamma \end{array}\right]=\left[\begin{array}{ccc} 0 & -\frac{\cos\psi }{\cos\vartheta } & \frac{\sin\gamma }{\cos\vartheta }\\ 0 & \sin\gamma & \cos\gamma \\ 1 & -\tan\vartheta \cos\gamma & \tan\vartheta \sin\gamma \end{array}\right]\left[\begin{array}{c} \Omega_{1}\\ \Omega_{2}\\ \Omega_{3} \end{array}\right]+\left[\begin{array}{ccc} \sin\psi \tan\vartheta & \cos\psi \tan\vartheta & -1\\ \cos\psi & -\sin\psi & 0\\ \frac{\sin\psi }{\cos\vartheta } & \frac{\cos\psi }{\cos\vartheta } & 0 \end{array}\right]\left[\begin{array}{c} \dot{\varphi }\\ -\left(u+\dot{\lambda }\right)\cos\varphi \\ -\left(u+\dot{\lambda }\right)\sin\varphi \end{array}\right].$$

The last expression (15) is a nonlinear differential homogeneous equation in which the unknowns are ψ, ϑ, and γ attitude angles. In expanded form Equation (15) can be rewritten as follows:

(16) $$\begin{array}{c} \dot{\psi }=-\Omega_{2}\frac{\cos\psi }{\cos\upsilon }+\Omega_{3}\frac{\sin\gamma }{\cos\upsilon }+\dot{\varphi }\sin\psi \tan\vartheta +\left(u+\dot{\lambda }\right)\left(\sin\varphi -\cos\varphi \cos\psi \tan\vartheta \right),\\ \dot{\vartheta }=\Omega_{2}\sin\gamma +\Omega_{3}\cos\gamma +\dot{\varphi }\cos\psi +\left(u+\dot{\lambda }\right)\cos\varphi \sin\psi ,\\ \dot{\gamma }=\Omega_{1}-\Omega_{2}\tan\vartheta \cos\gamma +\Omega_{3}\tan\vartheta \sin\gamma +\dot{\varphi }\frac{\sin\psi }{\cos\vartheta }-\left(u+\dot{\lambda }\right)\cos\varphi \frac{\cos\psi }{\cos\vartheta }. \end{array}$$

Systems of Equation (15) or (16) solutions can be obtained in the OC in following conditions:

1. $\Omega_{1,2,3}$ angular rate measurements are received by OC from sensors;
2. $\dot{\varphi }$, $\dot{\lambda }$, and φ values are computed in OC during navigation algorithm implementation;
3. ψ, ϑ, and γ angles initial values are either set up or determined during initialization.

The navigation algorithm mentioned above requires acceleration vector $\bar{n}$ projections on to the local level frame. In other words, the operation corresponding to the expression (4) should be performed in the OC. For that reason, it is necessary to calculate the $\left[C_{ij}\right]$ matrix, whose elements are determined using (2), and ψ, ϑ, and γ angles are calculated using (15) or (16).

Hence, the attitude determination algorithm and navigation algorithm are related as in the previous case of the $\left[C_{ij}\right]$ matrix calculation. Note, this relation will be removed if $\dot{\varphi }$, $\dot{\lambda }$, and φ values are received by OC from non-inertial system, for example GNSS. In this case, it will be an attitude determination algorithm implementing independently of the SINS attitude determination algorithm. In fact, it will be a system that can be called gyro-satellite or gyro-GNSS AHRS (Attitude and Heading Reference System).

## 3. Experimental Results

In this experimental investigation, there was an attempt to estimate advantages and disadvantages of different types of attitude determination functional algorithm implemented in inertial systems.

Four types of algorithm were investigated:

1. Algorithm based on differential Equation (3), Transformation matrix approach;
2. Algorithm based on differential Equation (15), Direct angles calculation approach;
3. Algorithm based on differential Equation (3) with $\Omega_{X,Y,Z}$ computed using GNSS velocity and coordinates data (gyro-GNSS AHRS), Transformation matrix approach using GNSS data;
4. Algorithm based on differential Equation (15) with $\Omega_{X,Y,Z}$ computed using GNSS velocity and coordinates data (gyro-GNSS AHRS), Direct angles calculation approach using GNSS data.

Implementation of algorithms based on the transformation matrix approach (types 1 and 3) requires orthogonalizaiton of the $\left[C_{ij}\right]$ matrix at each step of equation integration. Orthogonalization was carried out as follows:

$$\begin{array}{c} \left[D_{ij}\right]=0.5\left(\left[C_{ij}\right]{\left[C_{ij}\right]}^{T}-E\right),\\ \left[C_{ij}\right]=(E-\left[D_{ij}\right])\;\left[C_{ij}\right], \end{array}$$

where E is the identity matrix [3×3].

Experimental data were collected during car and helicopter tests.

### 3.1. Car Test

**Figure 2:**
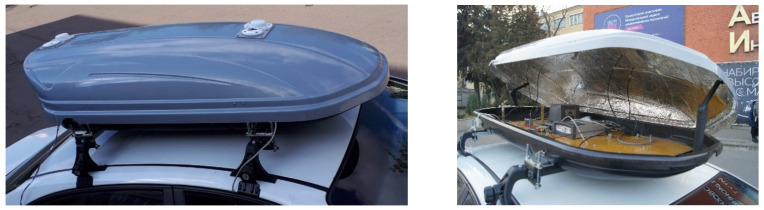
MAI Navigation System Car Testbed.

**Figure 3:**
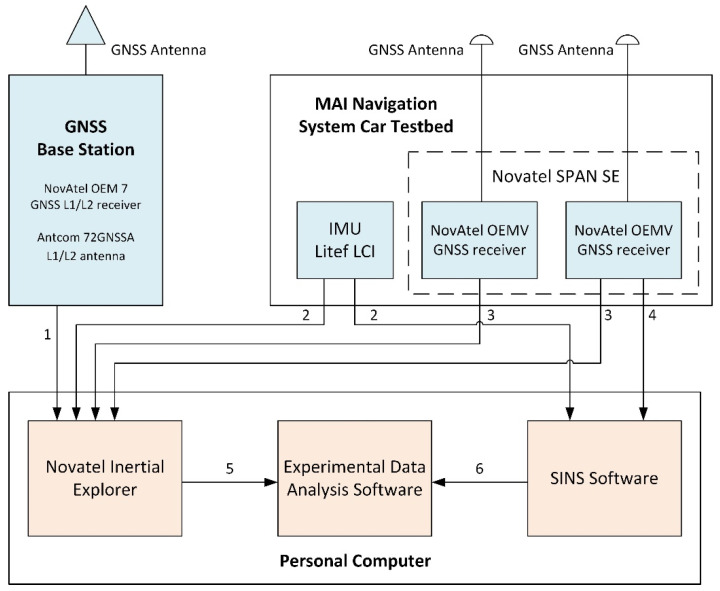
Car test experimental setup (1—GNSS Base Station Raw Measurements, 2—IMU measurements, 3—GNSS Rover Receivers Raw Measurements, 4—GNSS Navigation Data, 5—Reference Attitude Parameters, and 6—Investigated Attitude Parameters).

The car test was carried out with the use of an inertial measurement unit Litef LCI [13], based on fiber optic gyroscopes, and NovAtel OEMV GNSS receivers included in the MAI Navigation System Car Testbed installed inside a car roof rack [14] (Figure 2). The car test experimental setup is shown on Figure 3. Raw inertial data output rate was recorded at 200 Hz rate. The start time of the car test in GPS Time was 303,000 s, the end time was 315,000 s. Thus, the duration of the experiment was about 3.3 h.

**Figure 4:**
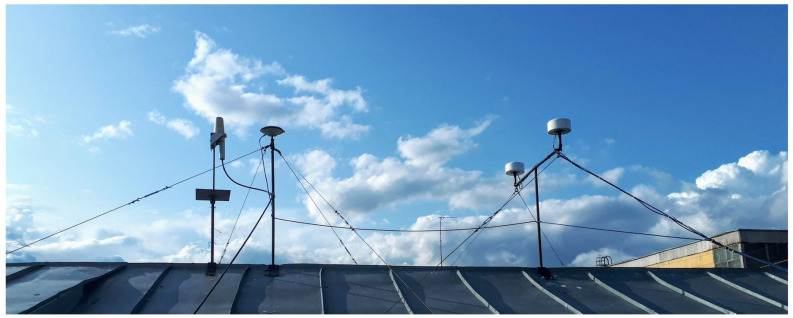
MAI GNSS laboratory antennas installed on the roof of a building.

**Figure 5:**
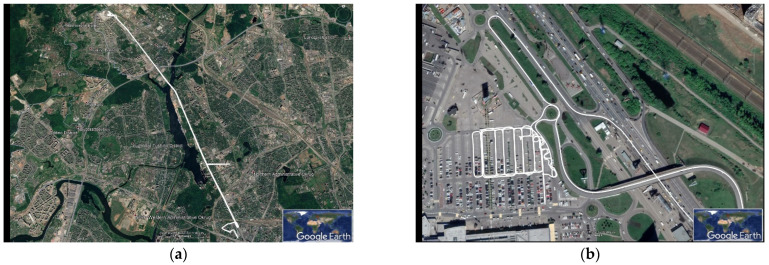
Car test track: (**a**) whole track; and (**b**) track leg with active maneuvering.

NovAtel Inertial Explorer (IE) software for postprocessing of INS and GNSS measurements was used to evaluate the reference attitude parameters [15]. For post-processing using IE INS and GNSS, measurements were also recorded from the IMU Litef LCI and two NovAtel OEMV GNSS L1/L2 receivers outputs that are the part of Novatel SPAN SE. The NovAtel OEM 7 GNSS L1/L2 receiver with Antcom 72GNSSA L1/L2 antenna installed on the roof of the MAI GNSS laboratory (Figure 4) was used as the GNSS base station. INS and GNSS measurements were processed in IE by Novatel tightly coupled (TC) algorithm that uses GPS carrier phase to limit error during periods where satellite tracking is limited or variable (even if only two or three satellites are visible). The possibility of using the TC algorithm is especially important when testing onboard a car in urban conditions. Car track is shown in Figure 5.

**Figure 6:**
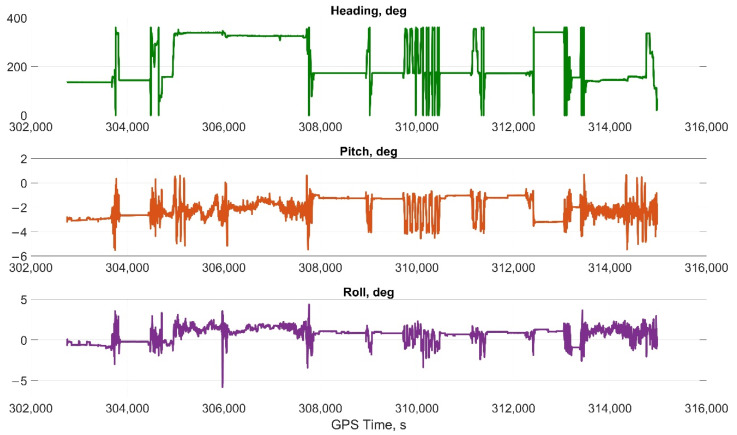
Reference values of the attitude angles in car test.

**Figure 7:**
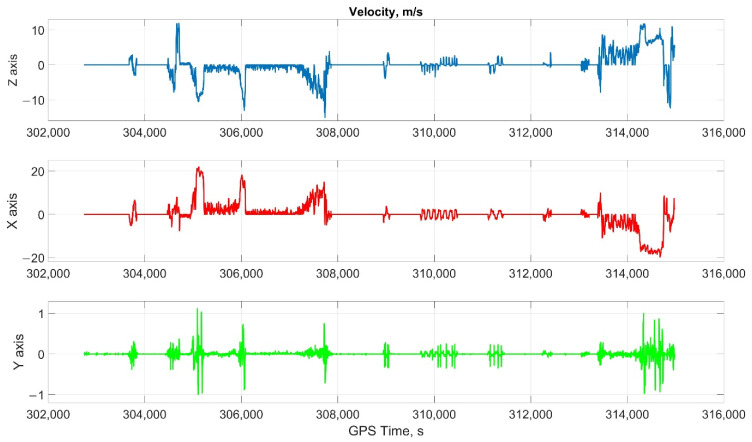
The north, east, and up velocity projections in car test.

**Figure 8:**
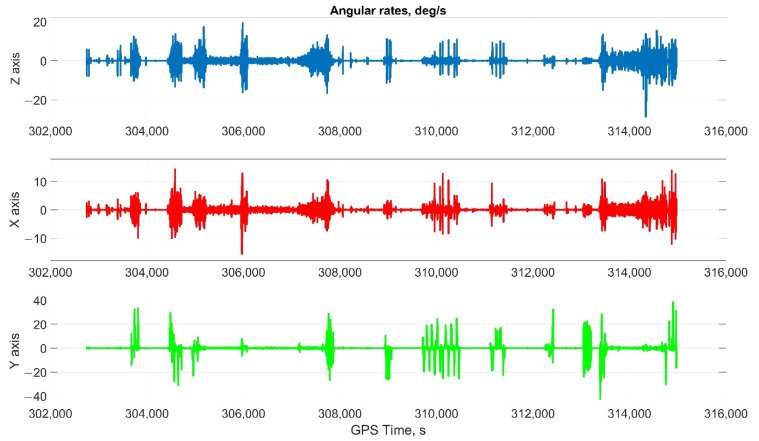
The angular rates in the projections onto body frame in car test (X, lateral axis; Y, longitudinal axis; and Z, completes the right-handed system, up axis when OXY is horizontal plane).

**Figure 9:**
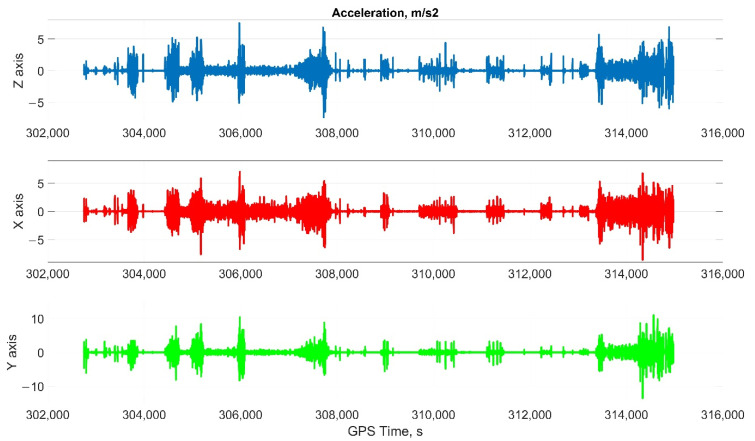
Accelerations in projections onto the body frame in car test (X, lateral axis; Y, longitudinal axis; and Z, completes the right-handed system, up axis when OXY is horizontal plane).

The car experiment was carried out with the motion parameters are shown in the following figures:

- The reference values of the attitude angles, Figure 6;
- The north, east and up velocity projections, Figure 7;
- The angular rates in the projections onto body frame, Figure 8;
- Accelerations in projections onto the body frame, Figure 9.

**Figure 10:**
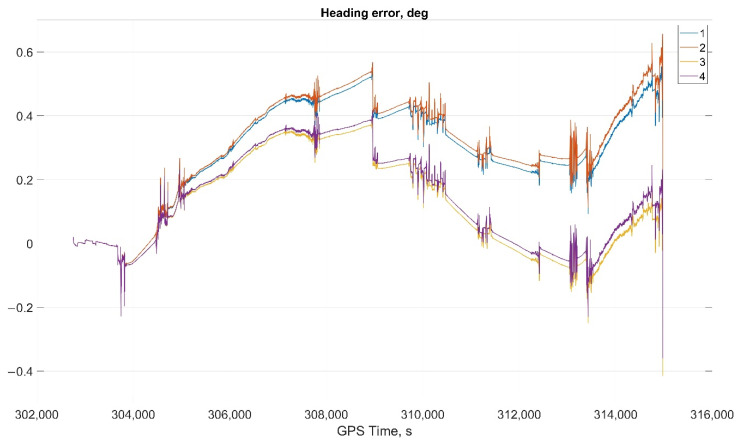
Heading error in car test (1, Transformation matrix approach; 2, Direct angles calculation approach; 3, Transformation matrix approach using GNSS data; and 4, Direct angles calculation approach using GNSS data.

**Figure 11:**
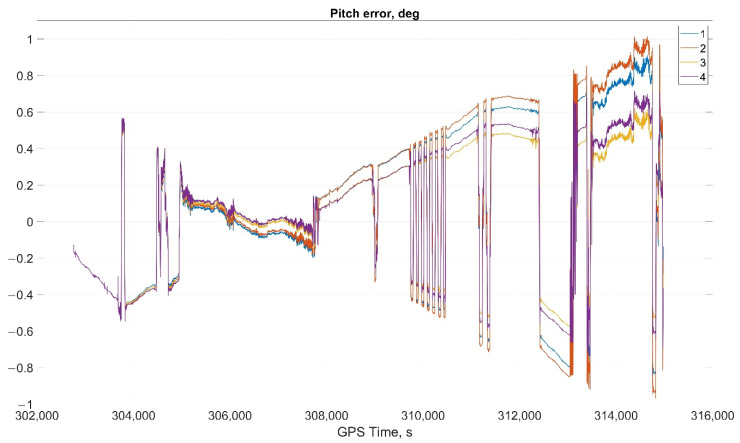
Pitch error in car test (1, Transformation matrix approach; 2, Direct angles calculation approach; 3, Transformation matrix approach using GNSS data; and 4, Direct angles calculation approach using GNSS data).

**Figure 12:**
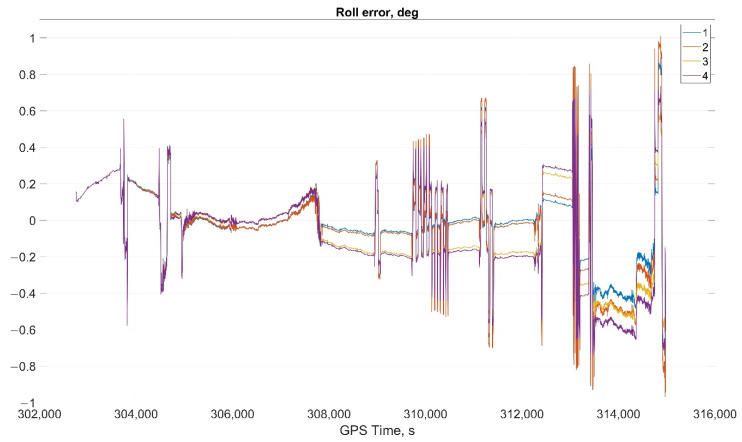
Roll error in car test (1, Transformation matrix approach; 2, Direct angles calculation approach; 3, Transformation matrix approach using GNSS data; and 4, Direct angles calculation approach using GNSS data).

The results of attitude parameters calculation in the car test using different types of algorithm are shown in Figure 10 (Heading error), Figure 11 (Pitch error), and Figure 12 (Roll error).

The comparison of attitude errors mean values and RMS are given in Table 1.

### 3.2. Helicopter Test

**Figure 13:**
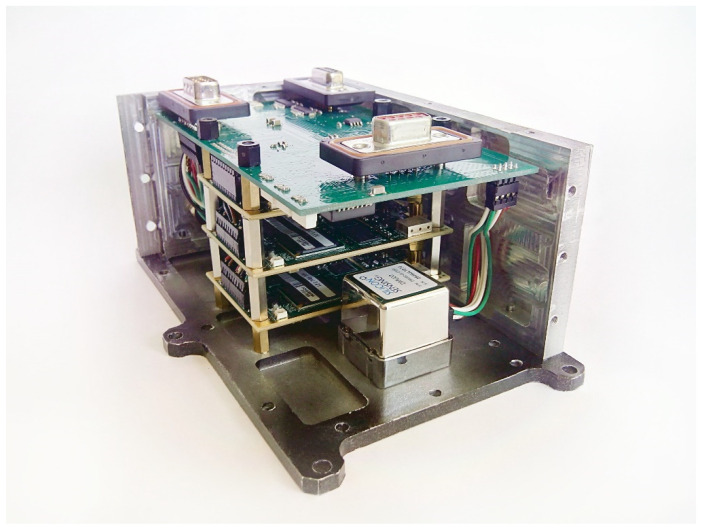
Small-sized integrated navigation system.

The helicopter test was carried out with the use of a small-sized integrated navigation system manufactured by MAI [16] (Figure 13), based on inertial module DMU02 [17] manufactured by Silicon Sensing, satellite navigation GLONASS/GPS receiver OEMV-1G [18] manufactured by NovAtel. Raw inertial data was recorded with 100 Hz rate.

**Figure 14:**
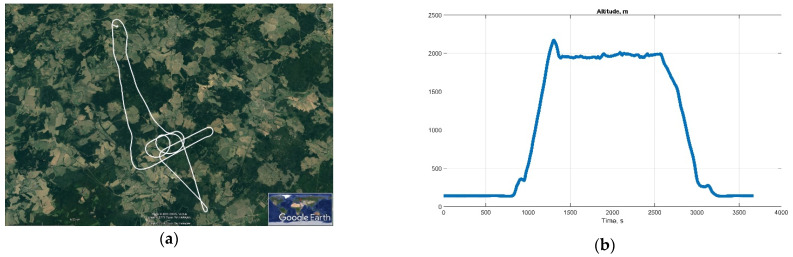
Helicopter test track: (**a**) horizontal track; and (**b**) altitude.

Reference attitude parameters were obtained from INS (Litef LCI), and GNSS (NovAtel OEMV GNSS) measurements postprocessed by NovAtel Inertial Explorer software. The helicopter test flight horizontal track and altitude are shown in Figure 14.

**Figure 15:**
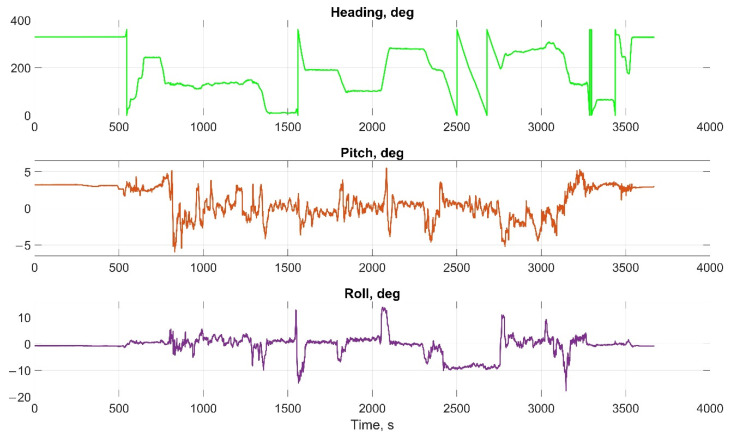
Reference values of the attitude angles in helicopter test.

**Figure 16:**
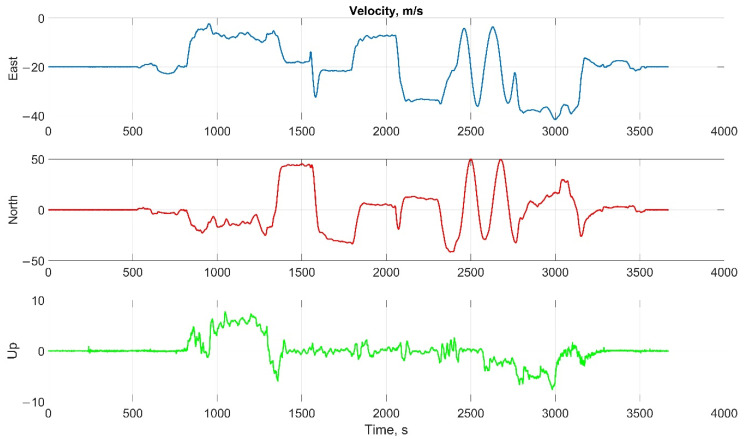
The north, east, and up velocity projections in helicopter test.

**Figure 17:**
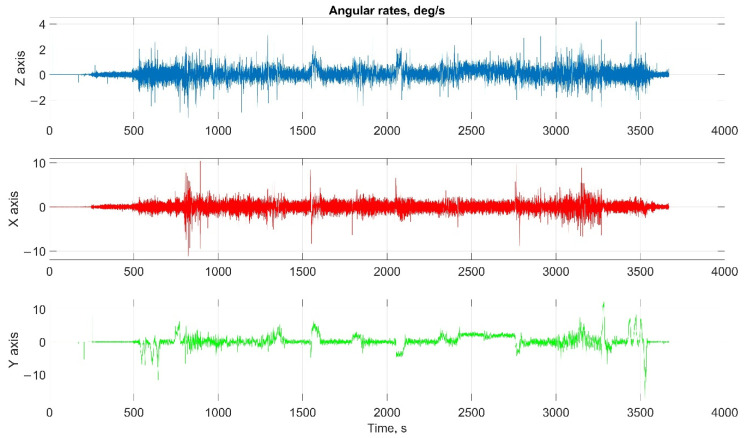
The angular rates in the projections onto body frame in helicopter test (X, lateral axis; Y, longitudinal axis; and Z, completes the right-handed system, up axis when OXY is horizontal plane).

**Figure 18:**
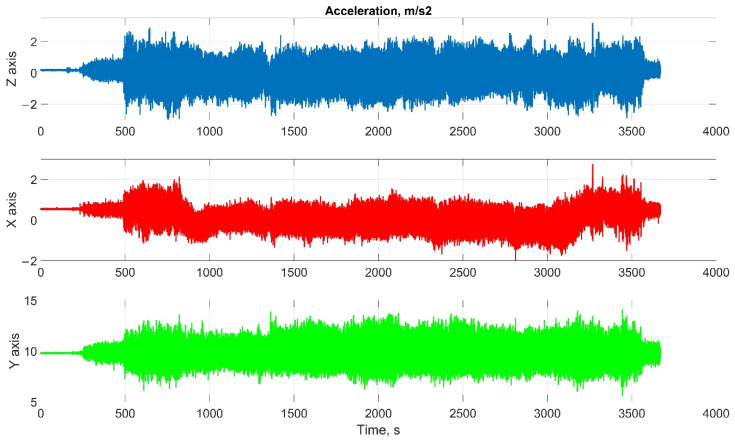
Accelerations in projections onto the body frame in helicopter test (X, lateral axis; Y, longitudinal axis; Z, completes the right-handed system, up axis when OXY is horizontal plane).

The helicopter experiment was carried out with the motion parameters are shown in the following figures:

- The reference values of the attitude angles, Figure 15;
- The north, east and up velocity projections, Figure 16;
- The angular rates in the projections onto the body frame, Figure 17;
- Accelerations in projections onto the body frame, Figure 18.

**Figure 19:**
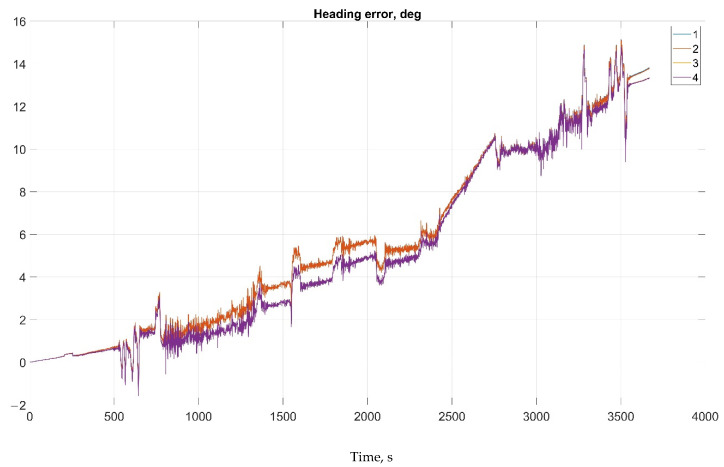
Heading error in helicopter test (1, Transformation matrix approach; 2, Direct angles calculation approach; 3, Transformation matrix approach using GNSS data; 4, Direct angles calculation approach using GNSS data).

**Figure 20:**
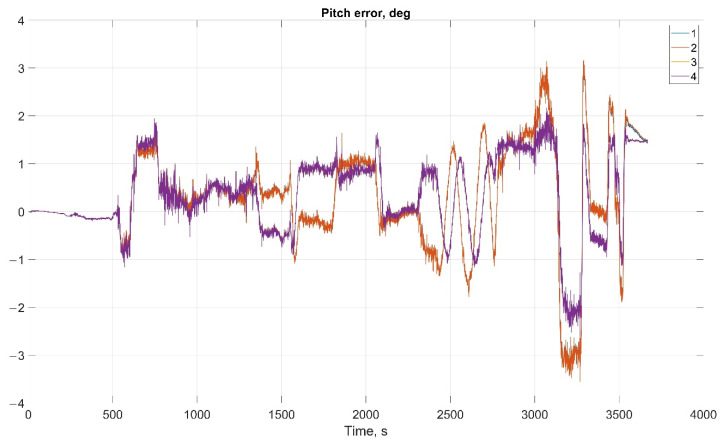
Pitch error in helicopter test (1, Transformation matrix approach; 2, Direct angles calculation approach; 3, Transformation matrix approach using GNSS data; 4, Direct angles calculation approach using GNSS data).

**Figure 21:**
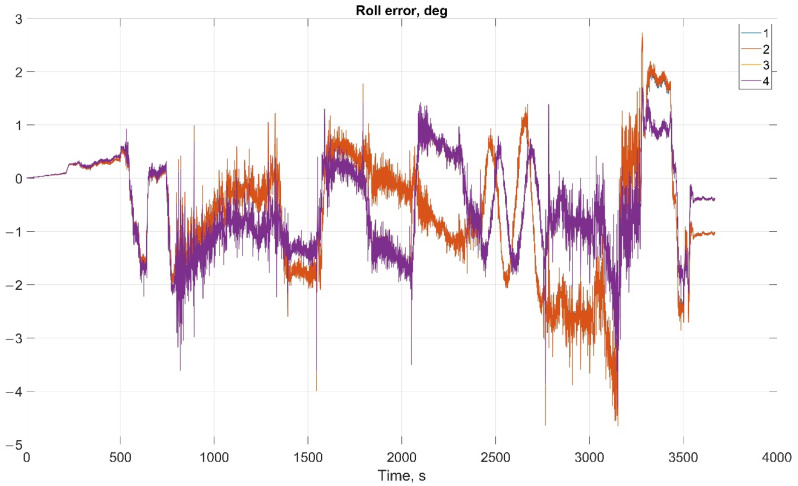
Roll error in helicopter test (1, Transformation matrix approach; 2, Direct angles calculation approach; 3, Transformation matrix approach using GNSS data; 4, Direct angles calculation approach using GNSS data).

The results of attitude parameters calculation in the car test using different types of algorithm are shown in Figure 19 (Heading error), Figure 20 (Pitch error), and Figure 21 (Roll error).

The lack of values in the “Heading, Mean” column is caused by the heading error increase.

## 4. Discussion

The qualitative differences between the direct attitude angles calculations algorithm and the one determining attitude using elements of the transformation matrix can be noted.

Obviously, decreasing the order of differential equations system from ninth to third can be attributed to positive qualities. The transformation matrix, whose elements are computed using previously determined ψ, ϑ, and γ angles, can be considered orthogonal.

Since scale and orthogonality errors occurring in equalities:

$$\begin{array}{c} C_{11}^{2}+C_{12}^{2}+C_{13}^{2}=C_{21}^{2}+C_{22}^{2}+C_{32}^{2}=C_{31}^{2}+C_{32}^{2}+C_{33}^{2}=1,\\ C_{11}^{2}C_{21}^{2}+C_{12}^{2}C_{22}^{2}+C_{13}^{2}C_{23}^{2}=C_{11}^{2}C_{31}^{2}+C_{12}^{2}C_{32}^{2}+C_{13}^{2}C_{33}^{2}=C_{21}^{2}C_{31}^{2}+C_{22}^{2}C_{32}^{2}+C_{23}^{2}C_{33}^{2}=0 \end{array}$$

will depend on the calculation accuracy of sines and cosines of attitude angles; these functions can be determined in the OC with the accuracy that allows to consider negligible this group of errors.

If the $\left[C_{ij}\right]$ matrix is determined by integrating the equations system (3), then the errors of scale and non-orthogonality depend on numerical method used in OC to solve this system.

Noteworthy, is one more feature of the direct attitude angles calculations algorithm that imposes restrictions on its application. The right-hand sides of equations systems (14) and (15) contain ϑ, tanϑ, and $\frac{1}{\cos\vartheta }$ angle functions. In aviation, for example, the limits of angle ϑ changes are usually accepted as ϑ=±90°. Obviously, these functions cannot be calculated in the OC in case of ϑ→90°. However, even for most aviation applications, pitch angles are limited and do not exceed 45°. The values of this angle for marine or automobile applications are significantly smaller.

Considering the error values experimental results shows the following. There are no advantages (Car test—Table 1, Figure 8, Figure 9 and Figure 10; and Helicopter test—Table 2, Figure 17, Figure 18 and Figure 19) of the direct attitude angles calculation algorithm based on differential Equation (15) compared to the algorithm of determining attitude using transformation matrix elements based on differential Equation (3) in test conditions described above. Such advantages may appear when inertial data output rate or attitude calculation rate due to low OC performance will be reduced. In this case, scale and orthogonality errors may occur in algorithm based on the transformation matrix approach. As expected, attitude determination errors using algorithms based on both approaches using GNSS velocity and coordinates data (gyro-GNSS AHRS) are smaller than the inertial-only solution. In current test conditions there are also no advantages of the direct attitude angles calculation algorithm over the algorithm of determining attitude using transformation matrix elements, even using GNSS velocity and coordinates data.

Special attention should be paid to the description of the heading error results obtained during the helicopter test (Figure 19). A significant error increase is explained by the low accuracy of MEMS gyroscopes. Moreover, such error levels occur both in the autonomous SINS and gyro-GNSS AHRS modes, since satellite data is used only to calculate the position of local-level frame origin. In this case, the heading error mainly depends on the accuracy of measuring the object rotation around the vertical axis. These measurements are carried out by gyroscopes.

Comparing the achieved accuracy of calculating the attitude parameters with the results of other studies, the following can be concluded.

The performance comparison analysis of FOGs and MEMS IMUs under an enhanced GPS/Reduced INS land vehicles navigation system is given in [19]. Novatel IMU-CPT was used as the FOGs IMU. As part of the tests, among other things, the accuracy of determining the attitude parameters during a GPS outage was estimated. Two trajectories in urban conditions with velocities of 50–60 km/h with stops were chosen to carry out the research. The accuracy of determining the heading angle in the work [19] was at the levels from 0.5 to 2.1 degrees (RMS), which is somewhat rougher than the results given in Table 1 (0.2–0.4 degrees). The accuracy of the roll/pitch determination in the work [19] is the level from 0.5 to 3 degrees (RMS). The accuracy of the roll/pitch determination in Table 1 is about 0.2 degrees.

The authors of [20] present the results of Optolink FOG-based SINS tests. The marine system Optolink SINS500M was subjected to tests on a car, rotary test bench, and ships. The characteristics of FOG-based SINSs produced by Optolink and the leading world manufacturers are also given in [20]. A system of a similar accuracy level (LISA200 Northrop Grumman, FOG bias drift is less than 0.5 deg/h) has pitch/roll error of 0.3 degrees, and heading error of 0.8 degrees. The angle determination accuracy obtained during tests on a car with Litef LCI (FOG bias drift is less than 1.0 deg/h) was comparable and even higher (Table 1).

The results of experimental studies on velocity-aided attitude estimation for helicopter aircraft using microelectromechanical system inertial-measurement units are described in [21]. At the same time, the accuracy of the autonomous attitude determination was separately estimated. The analog device ADIS 16488 was used as an IMU. The tests were carried out on a Korean utility helicopter (Surion). A flight test profile was conducted in accordance with an acceptance test procedure comprising a series of steps: take off, roll maneuver, pitch maneuver, landing, take off, loitering flight, and landing. The accuracy estimation was carried out only for roll and pitch angles. The error ranged from 0.33 to 0.41 degrees (RMS). The errors in determining these angles given in Table 2 are at the levels from 0.8 to 1.1 degrees. Possible reasons for the obtained results showed the lower accuracy are different IMU characteristics, and differences in the helicopter trajectory parameters, as well as the implementation in [21] of the improved attitude determination algorithm using the estimation of disturbance in the accelerometer measurements. The estimation of disturbance in the accelerometer measurements is carried out by the airspeed obtained from the air data computer.

## 5. Conclusions

The article described an approach to the derivation of attitude determination algorithm equations in strapdown inertial navigation system. This algorithm is based on direct heading, pitch, and roll calculation. The analysis of an algorithm implementing the derived equations is based on its comparison with the transformation matrix-based algorithm when both of them are implemented in the INS during car and helicopter tests.

Comparison of the obtained results with the results of tests of similar IMUs on similar types of moving objects suggests that the developed and implemented attitude algorithms provide the same, or even better, accuracy than the algorithms described above.

Further research requires the development of direct attitude angles calculation error equations for simulation study of the various factors influence on attitude angles determination errors.

## Figures and Tables

**Table 1:** Attitude errors mean values and RMS in car test.

Algorithm Type	Heading, Deg.		Pitch, Deg.		Roll, Deg.	
	Mean	RMS	Mean	RMS	Mean	RMS
1 ^1^	0.29	0.16	0.11	0.43	−0.02	0.21
2 ^2^	0.30	0.16	0.13	0.47	−0.03	0.23
3 ^3^	0.12	0.14	0.07	0.32	−0.05	0.23
4 ^4^	0.14	0.15	0.08	0.35	−0.07	0.26

^1^ Transformation matrix approach, ^2^ Direct angles calculation approach, ^3^ Transformation matrix approach using GNSS data, and ^4^ Direct angles calculation approach using GNSS data.

**Table 2:** Attitude errors mean values and RMS in helicopter test.

Algorithm Type	Heading, Deg.		Pitch, Deg.		Roll, Deg.	
	Mean	RMS	Mean	RMS	Mean	RMS
1 ^1^	-	4.26	0.29	1.02	−0.60	1.13
2 ^2^	-	4.26	0.29	1.04	−0.60	1.14
3 ^3^	-	4.31	0.35	0.82	−0.51	0.84
4 ^4^	-	4.31	0.35	0.82	−0.51	0.84

^1^ Transformation matrix approach, ^2^ Direct angles calculation approach, ^3^ Transformation matrix approach using GNSS data, and ^4^ Direct angles calculation approach using GNSS data.
